# Dextromethorphan Inhibits Activations and Functions in Dendritic Cells

**DOI:** 10.1155/2013/125643

**Published:** 2013-05-28

**Authors:** Der-Yuan Chen, Pei-Shan Song, Jau-Shyong Hong, Ching-Liang Chu, I-Horng Pan, Yi-Ming Chen, Ching-Hsiung Lin, Sheng-Hao Lin, Chi-Chen Lin

**Affiliations:** ^1^Institute of Biomedical Science, National Chung-Hsing University, Taichung 402, Taiwan; ^2^Faculty of Medicine, National Yang-Ming University, Taipei 112, Taiwan; ^3^Division of Allergy, Immunology and Rheumatology, Taichung Veterans General Hospital, Taichung 407, Taiwan; ^4^Laboratory of Toxicology and Pharmacology, National Institutes of Environmental, Health Sciences, Research Triangle Park, NC 27709, USA; ^5^Graduate Institute of Immunology, College of Medicine, National Taiwan University, Taipei 100, Taiwan; ^6^Biomedical Technology and Device Research Laboratories, Industrial Technology Research Institute, Hsinchu 300, Taiwan; ^7^Institute of Clinical Medicine, National Yang-Ming University, Taipei 112, Taiwan; ^8^Division of Chest Medicine, Department of Internal Medicine, Changhua Christian Hospital, Changhua 500, Taiwan; ^9^Department of Respiratory Care, College of Health Sciences, Chang Jung Christian University, Tainan 711, Taiwan; ^10^School of Medicine, Chung Shan Medical University, Taichung 402, Taiwan; ^11^Department of Medical Research and Education, Taichung Veterans General Hospital, Taichung 407, Taiwan

## Abstract

Dendritic cells (DCs) play an important role in connecting innate and adaptive immunity. Thus, DCs have been regarded as a major target for the development of immunomodulators. In this study, we examined the effect of dextromethorphan (DXM), a common cough suppressant with a high safety profile, on the activation and function of DCs. In the presence of DXM, the LPS-induced expression of the costimulatory molecules in murine bone marrow-derived dendritic cells (BMDCs) was significantly suppressed. In addition, DXM treatment reduced the production of reactive oxygen species (ROS), proinflammatory cytokines, and chemokines in maturing BMDCs that were activated by LPS. Therefore, DXM abrogated the ability of LPS-stimulated DCs to induce Ag-specific T-cell activation, as determined by their decreased proliferation and IFN-**γ** secretion in mixed leukocyte cultures. Moreover, the inhibition of LPS-induced MAPK activation and NF-**κ**B translocation may contribute to the suppressive effect of DXM on BMDCs. Remarkably, DXM decreased the LPS-induced surface expression of CD80, CD83, and HLA-DR and the secretion of IL-6 and IL-12 in human monocyte-derived dendritic cells (MDDCs). These findings provide a new insight into the impact of DXM treatment on DCs and suggest that DXM has the potential to be used in treating DC-related acute and chronic diseases.

## 1. Introduction

Dendritic cells (DCs), a highly specialized type of bone marrow-derived leukocytes that are important for the initiation of T-cell responses, link innate and adaptive immunity. They are present in different stages of maturation in the circulation as well as in lymphoid and nonlymphoid organs. DCs reside in an immature form in nonlymphoid tissues, where they act as sentinels [1–3]. After they capture and process antigens in peripheral nonlymphoid tissues, DCs migrate through afferent lymph to the T-cell-dependent areas of secondary lymphoid organs (e.g., lymph nodes), where they activate naive T-cell responses and undergo phenotypic and functional changes (i.e., maturation). The immunostimulatory properties of mature DCs include increased surface expression of major histocompatibility complexes (MHCs) with Ag-peptides and costimulatory molecules (e.g., CD40, CD80), increased secretion of cytokines and chemokines, and reduced Ag uptake [4, 5]. While mature DCs can potently initiate primary T-cell-mediated immune responses, immature DCs stimulate T-cell responses only weakly or may even promote the generation of regulatory T (Treg) cells [6]. 

Because pharmacological modulation of DC activation prevents the development of several T-cell-mediated diseases [7], DCs may represent a new therapeutic approach for treating harmful immune responses such as hypersensitivity reactions and autoimmunity [8, 9]. Notably, the clinical efficacy of corticosteroids and other antirheumatic drugs, such as gold sodium thiomalate, leflunomide, mycophenolic acid, and valproic acid, may be due to their significant disruption of DC function [10–15].

Dextromethorphan (d-3-methoxy-17-methylmorphinan, abbreviated DXM), a dextrorotatory morphinan, is widely and clinically used as an antitussive. There is an increasing evidence that DXM has anti-inflammatory and immunomodulatory effects. DXM protects mice against lipopolysaccharide/GalN-induced endotoxemia and liver damage; the mechanism of protection may involve faster TNF-*α* clearance, decreased superoxide production, and decreased expression of genes associated with inflammation and hepatocellular death [16]. In addition, DXM prevents moderate experimental autoimmune encephalomyelitis by inhibiting the NOX2-mediated production of ROS and decreasing the infiltration of monocytes and lymphocytes into the spinal cord [17]. DXM reduces Group A Streptococcal (GAS)-induced systemic inflammatory responses and organ injury in mice [18]. Furthermore, DXM reduces cytokine and superoxide production in macrophages by inhibiting NAPDH oxidase, resulting in decreased atherosclerosis and neointima in mice [19]. DXM attenuates oxidative stress and inflammation markers in habitual smokers [20]. Because the cellular targets of DXM in the immune system have yet to be studied, the role of DXM in the cellular maturation and immunoregulatory activity of DCs is an open question.

In this study, we examined the potential effects of DXM on the maturation and functional properties of DCs. We found that DXM inhibited the LPS-induced functional maturation of murine BMDCs and human MDDCs. In addition, DXM downregulated the LPS-induced MAPK signaling pathways (ERK1/2, JNK, and p38 MAPK), I*κ*B expression, and NF-*κ*Bp65 nuclear translocation. Taken together, these results suggest that DXM manipulates the immunostimulatory properties of DC and may have important applications against harmful immune responses such as chronic inflammation, autoimmunity, and transplantation.

## 2. Material and Methods

### 2.1. Mice and Preparation of Bone Marrow-Derived Murine DCs

Five- to eight-week-old specific pathogen-free female C57BL/6 (H-2^b^) mice were purchased from the National Laboratory Animal Center (Taipei, Taiwan) or the National Cheng-Kung University (Tainan, Taiwan). OT-I TCR transgenic mice were purchased from Jackson Lab (Bar Harbor, ME, USA), and OT-II TCR transgenic mice were provided by Dr. Clifford Lowell (UCSF, San Francisco, CA, USA). All mice were housed in the barrier facility at Taichung Veterans General Hospital (Taichung, Taiwan) in accordance with the Institutional Animal Care and Use Committee guidelines for animal experimentation. Murine bone marrow-derived DCs were generated as previously described [21]. Briefly, femurs and tibias were aseptically removed from mice. After the surrounding muscle tissue was removed, the bones were placed in a 10 mm dish with 70% alcohol for 1 min, washed twice with phosphate-buffered saline (PBS), and transferred into a fresh dish with RPMI 1640 medium. Both ends of the bones were cut with scissors, and the marrow was flushed with RPMI 1640 using a syringe and a 25-gauge needle. The red cells were lysed with ammonium chloride. Bone marrow cells (5–7 × 10^5^ cells/mL) were suspended in RPMI-1640 supplemented with 10% heat-inactivated fetal bovine serum, 2 mm L-glutamine, 100 U/mL penicillin, 100 *μ*g/mL streptomycin, 5 × 10^5^ M 2-ME, 10 mM HEPES (pH 7.4), 20 ng/mL recombinant murine granulocyte macrophage colony-stimulating factor (PeproTech), and 20 ng/mL recombinant murine IL-4 (PeproTech). Cells were placed in 6-well plates. The culture medium was changed every 2 days, and nonadherent or loosely adherent cells were harvested on day 7 and used as immature DCs. More than 80% of the cells expressed CD11c, as determined using flow cytometry. CD11c^+^ DCs were further selected from BM cells with CD11c (N418) microbeads (Miltenyi Biotec), according to the manufacturer's instructions, and these cells were used for the experiments. The purity of the CD11c^+^ cells was >90% (data not shown). 

### 2.2. Generation of Human Monocyte-Derived DCs

DCs were prepared from peripheral blood monocytes (PBMCs) by standard procedures. Briefly, peripheral blood was collected from healthy volunteer donors, and PBMCs were isolated from peripheral blood buffy coats by magnetic cell sorting with anti-CD14 MicroBeads, per the manufacturer's protocol (Miltenyi Biotec). The purity of the CD14+ fraction was always >90%, as assessed using flow cytometry. Purified monocytes were seeded in 6-well plates and cultured in complete medium ( RPMI 1640 (Gibco) containing 10% FBS (Gibco)), recombinant human 80 ng mL^−1^ GM-CSF (PeproTech), and 100 ng mL^−1^ IL-4 (PeproTech) to generate immature DCs. Every two days, fresh medium containing GM-CSF and IL-4 was added to the cells. After 7 days of culture, nonadherent or loosely adherent cells were harvested, washed once with PBS, and used for the experiments.

### 2.3. Flow Cytometry Analysis

The expression of cell surface molecules was quantified by flow cytometry as follows. DXM hydrobromide hydrate was purchased from Sigma-Aldrich, and a 12.5 mM stock solution was made with PBS. Aliquots of 2 × 10^5^ immature BMDCs or MDDCs were cultured in the presence or absence of DXM for 1 h and then stimulated with 100 ng/mL *Escherichia coli* serotype O26:B6 LPS (Sigma) or 100 ng/mL LPS plus 10 ng/mL IFN-*γ* (PeproTech) for 18 h. The control group was treated with PBS alone. After incubation, DCs were harvested and stained with the following antibodies for 45 min on ice (1 *μ*g/mL diluted in PBS/1.0% FCS (v/v)): FITC-conjugated anti-human CD1a^+^ or anti-murine CD11c^+^; phycoerythrin (PE)-conjugated anti-human CD80^+^, anti-human CD83^+^, anti-murine CD40, anti-murine CD80, anti-murine CD86, anti-murine MHC class I, anti-murine MHC class II or isotype-matched control mAbs (all of the above from Biolegends); or PE-conjugated anti-human HLA-DR (BD Pharmingen). After washing with PBS, the cells were analyzed in a FACSCalibur flow cytometer (BD Biosciences), and the data were analyzed using WINMDI software (Scripps, La Jolla, CA, USA).

### 2.4. Cytokine Assay

Supernatants were collected from DCs (1 × 10^6^/mL) propagated in the presence or absence of DXM for 1 h. The cells were then stimulated with 100 ng/mL LPS or 100 ng/mL LPS plus 10 ng/mL IFN-*γ* or other TLR ligands, including Pam3CSK4 (5 *μ*g/mL, TLR1/TLR2), PolyI:C (250 *μ*g/mL, TLR3), flagellin (500 ng/mL, TLR5), and CpG ODN 1826 (200 nM TLR-9) (all from InvivoGen) for 18 h (6 h for TNF-alpha and RANTES). The control group was treated with PBS alone. After incubation, cytokine and chemokine production in DC supernatants was determined using sandwich ELISA assays, according to the manufacturer's specifications (PeproTech). 

### 2.5. Measurement of Reactive Oxygen Species (ROS)

ROS generation was measured after staining the cells with the oxidative sensitive dye 5-(and-6)-carboxy-2′,7′-dichlorodihydrofluorescein diacetate (DCFDA, Molecular Probes). For this assay, DC cells at a density of 3 × 10^5^ cells/mL were cultured in the presence or absence of DXM (50 *μ*M) for 1 h followed by stimulation with 100 ng/mL LPS. The control group was treated with PBS alone. After LPS stimulation for 6 h, the medium was removed, and culture medium containing 5 *μ*M DCFDA was added under low-light conditions. The cells were incubated for 30 min at 37°C, and the amount of ROS was analyzed by flow cytometry as described above.

### 2.6. DXM Cytotoxicity Assay

To determine cell viability and apoptosis, murine BMDCs and human MDDCs (2 × 10^5^ cells/mL) were cultured in the presence or absence of DXM for 1 h and stimulated with 100 ng/mL LPS for 18 h. The control group was treated with PBS alone. Cell viability was determined using the CCK-8 colorimetric assay according to the manufacturer's instructions (Sigma). To detect apoptosis, cell death was measured by flow cytometry using a phycoerythrin-conjugated Annexin V detection kit-I (BioVision) per the manufacturer's protocol. Flow cytometry was performed as described above by gating on 2 × 10^4^ CD11c^+^ DC cells per sample.

### 2.7. OVA-Specific T-Cell Activation

The protocol was modified from our previous report [22]. Briefly, purified DCs were pulsed with 2 *μ*g/mL OVA_257−264_ (OVAP_1_) or OVA_323−339_ (OVAP_2_) (synthesized by Echo Chemical Co., Taiwan) and incubated with LPS (100 ng/mL), DXM (50 *μ*M), or LPS plus DXM for 18 h. After incubation, the cells were harvested and washed with PBS. OVAP_1_ specific CD8^+^ T cells and OVAP_2_ specific CD4^+^ T cells were positively enriched from the spleens of OT-1 and OT-2 mice using the EasySep Murine CD8a or CD4 positive selection kits, respectively, according to the manufacturer's protocols (stem cells). The cells were more than 90% pure, as determined by flow cytometry with FITC-conjugated anti-CD4 and CD8 mAbs (Biolegends). Purified T cells (2 × 10^5^) and different treated DCs were added at various DC : T-cell ratios to 96-well round-bottom plates. After 3 days, T-cell proliferation was measured. [^3^H] thymidine (1 *μ*Ci; GE Healthcare) was added to the culture, and after an overnight incubation period, the incorporated [^3^H] thymidine was quantified by liquid scintillation counting (*β*-Counter; Beckman). In addition, supernatants from the DC—OT-I/OT-II cultures were collected after 3 days, and their IFN-*γ* levels were measured using an ELISA kit (eBioscience).

### 2.8. Preparation of Nuclear Extracts and Western Blot Analysis

Briefly, purified DCs were cultured in the presence or absence of 50 *μ*M DXM for 1 h and stimulated with LPS (100 ng/mL). Whole-cell lysates were prepared at the indicated time points, as described previously [23]. Nuclear extracts were prepared using the NE-PER nuclear and cytoplasmic Extraction system (Pierce), per the manufacturer's instructions. All of the steps in the preparations included the protease inhibitors leupeptin (Sigma-Aldrich) and aprotinin (Sigma-Aldrich) at 10 *μ*g/mL. Protein concentrations were determined using a BCA protein assay kit (Pierce). Protein extracts were boiled, resolved by SDS-PAGE and electrotransferred to nitrocellulose membranes. After blocking in 10% milk in TBS, the membranes were incubated with antibodies for phospho-p38 (Thr180/Tyr182), p38, phospho-p42/44 (Thr202/Tyr204, 20G11), total p42/44 (137F5), phosphor-JNK (81E11), JNK, anti-I*κ*B (56G8), anti- NF-*κ*B p65 (C22B4) (all purchased from cell signaling) or anti-Lamin B (M-20), anti-IDO Ab (mIDO-48) were purchased from Santa Cruz Biotechnology. The membranes were then washed, incubated with horseradish peroxidase-labeled secondary Abs (Jackson ImmunoResearch, West Grove, PA, USA), developed with enhanced chemoluminescence (Amersham), and analyzed with the LAS3000 system (Fujifilm, Tokyo, Japan). Densitometric analysis was performed with ImageJ software (National Institute of Health, Bethesda, MD, USA).

### 2.9. Statistical Analysis

The results are expressed as the mean ± SD. Statistical analyses were performed by one-way ANOVA, followed by Tukey's post-hoc test (Graphpad Prism 4.0, GraphPad Software). *P* values < 0.05 were considered statistically significant. 

## 3. Results

### 3.1. DXM Affects the Expression of Cell Surface Molecules in LPS-Stimulated Murine BMDCs

**Figure 1:**
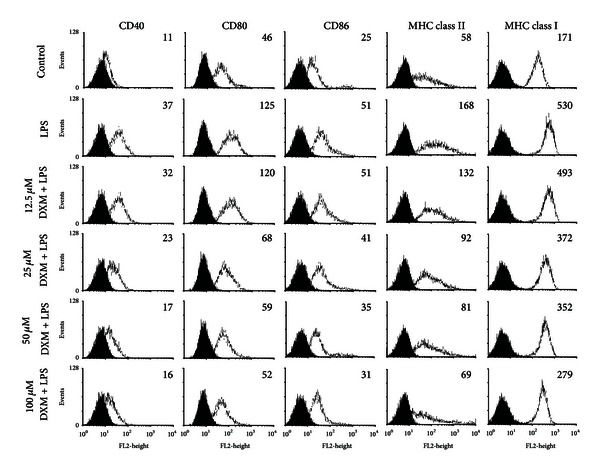
DXM reduces the expression of immunomodulatory cell surface markers in LPS-induced BMDCs. Immature BMDCs were stimulated with 100 ng/mL LPS with or without DXM for 18 h. Control groups were treated with PBS alone. After incubation, the expression of the surface markers CD40, CD80, CD86, MHC class I and MHC class II was analyzed by flow cytometry with fluorescently labeled Abs. The gray-filled area represents staining with an isotype-matched control Ab. The geometric mean fluorescence intensity (GMFI) of LPS or LPS+DXM is indicated. All data are representative of three independent experiments showing similar results.

In the first series of experiments, we investigated the effects of DXM on the maturation of immature DCs. Immature BMDCs were cultured in the presence of DXM (12.5, 25, 50, and 100 *μ*M) and then exposed to bacterial LPS which is a strong inducer of DC maturation. In general, DC maturation is accompanied by the enhanced expression of surface molecules, including costimulatory molecules and major histocompatibility complex molecules (MHC) that mediate adhesion with T cells by stabilizing the DC/T cells contact zone. Consistent with previously published data, the LPS stimulation of BMDCs resulted in the significant upregulation of costimulatory molecules (CD80, CD86 and CD40) and major histocompatibility complex molecules (MHC class II, MHC class I) within 18 h. While the DXM inhibition of LPS-induced maturation was dose-dependent, the expression of CD80, CD86, CD40, MHC class I, and MHC class II was significantly lower in the presence of DXM than in untreated mature BMDCs cells (Figure 1). These effects were not due to an increase in the number of dead cells (as determined by CCK-8 or flow cytometry with Annexin V); there was no marked difference in the percentage of dead cells in cultures containing 100 *μ*M DXM or PBS-treated controls (Supplemental Figure 1(a) available online at http://dx.doi.org/10.1155/2013/125643). These observations suggested that DXM impaired LPS-induced DC phenotypic maturation. 

### 3.2. DXM Modulates Cytokine, Chemokine, and ROS Production in LPS-Stimulated BMDCs

**Figure 2:**
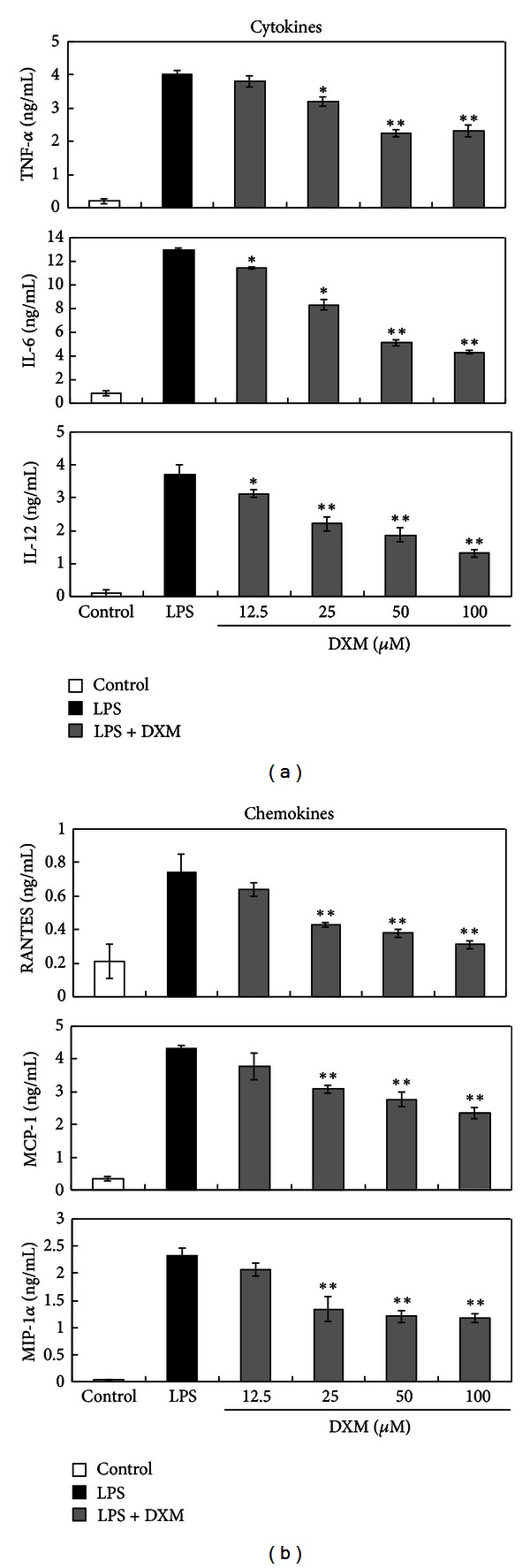
DXM impaired the release of cytokines and chemokines from LPS-stimulated BMDCs. Immature BMDCs were stimulated with 100 ng/mL LPS with or without DXM. The control group was treated with PBS alone. Culture supernatants were collected after 18 h (4 h for TNF-alpha and RANTES), and cytokines and chemokines were quantified by ELISA. Data are presented as the means ± SD of samples from three wells. Significant differences between DXM-treated and untreated LPS-activated BMDCs are shown with asterisks (*P < 0.05, **P < 0.01). All data are representative of three independent experiments.

**Figure 3:**
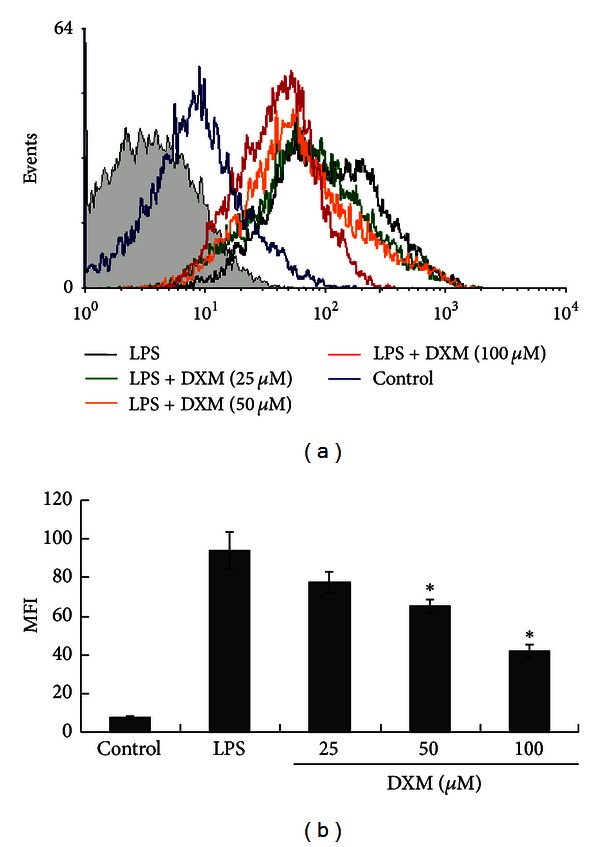
ROS production in LPS-stimulated BMDCs was impaired by DXM. Immature BMDCs were stimulated with 100 ng/mL LPS with or without DXM for 18 h. The control group was treated with PBS alone. After incubation, the cells were harvested, stained with DCFDA, and analyzed by flow cytometry. The mean fluorescence intensities for ROS generation were tabulated. The data are represented as the mean ± SD in triplicate tests. All data are representative of three independent experiments. Significant differences between DXM-treated and untreated LPS-activated BMDCs are shown with asterisks (*P < 0.05).

Mature DCs secrete cytokines and chemokines that modulate inflammatory responses and adaptive immunity [24]. We examined whether DXM altered cytokine and chemokine secretion in LPS-stimulated BMDCs. First, we examined changes in the BMDC TNF-alpha production, which is a hallmark of DC activation. TNF-alpha was quantified using ELISA for supernatants that were collected from LPS-triggered BMDCs propagated in the presence or absence of DXM.  Figure 2 shows that unstimulated immature BMDCs did not produce detectable levels of TNF-alpha. As expected, BMDCs started producing a large amount of TNF-alpha after stimulation with LPS, but DXM pretreatment led to dose-dependent significant decreases in TNF-alpha production. The secretion of other proinflammatory cytokines (e.g., IL-6, IL-12) and chemokines (e.g., MCP-1, MIP-1 alpha, and RANTES) was also inhibited by DXM. IL-12 production is an important marker for DC maturation and can be used to select Th1-dominant adjuvants (Figure 2). Additionally, increased levels of reactive oxygen species (ROS) are involved in the activation of DCs by different stimuli, and antioxidants inhibit DC activation [25]. To assess the potential intracellular mechanisms for DXM inhibition of DC maturation, we analyzed ROS levels in BMDCs pretreated with DXM and matured with LPS, which is known to increase ROS in DCs [25]. As expected, ROS levels were increased following treatment with LPS (Figure 3). However, treatment with DXM reduced LPS-induced ROS in BMDCs. These results further suggest that DXM attenuates the maturation and immunostimulatory activity of DCs activated by LPS.

### 3.3. DXM Inhibits the Ability of LPS-Stimulated BMDCs to Stimulate OVA-Specific T-Cell Proliferation

**Figure 4:**
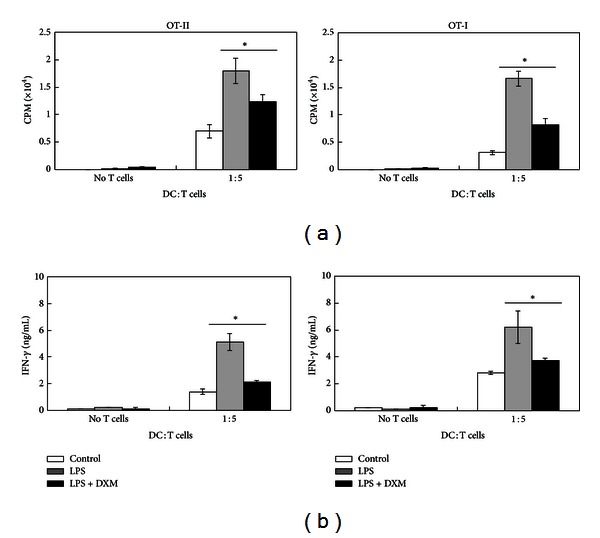
DXM inhibits Ag-specific T-cell activation by LPS-stimulated BMDCs. (a) Either OT-I CD8^+^ T cells or OT-II CD4^+^ T cells were cocultured with BMDCs pulsed with OVA peptide and treated with PBS, LPS (100 ng/mL) + PBS, or LPS + DXM (50 *μ*M) at the indicated ratio of DC : T cells for 3 days. The cells were exposed to [^3^H]-thymidine for 18 h before cell-associated radioactivity was determined. (b) Supernatants were collected from cultures after 4 days. IFN-*γ* production was measured by ELISA. The data shown are the mean ± SD of samples of three wells. Significant differences between DXM-treated and untreated LPS-activated BMDCs are shown with asterisks (*P < 0.05). All data are representative of three independent experiments.

**Figure 5:**
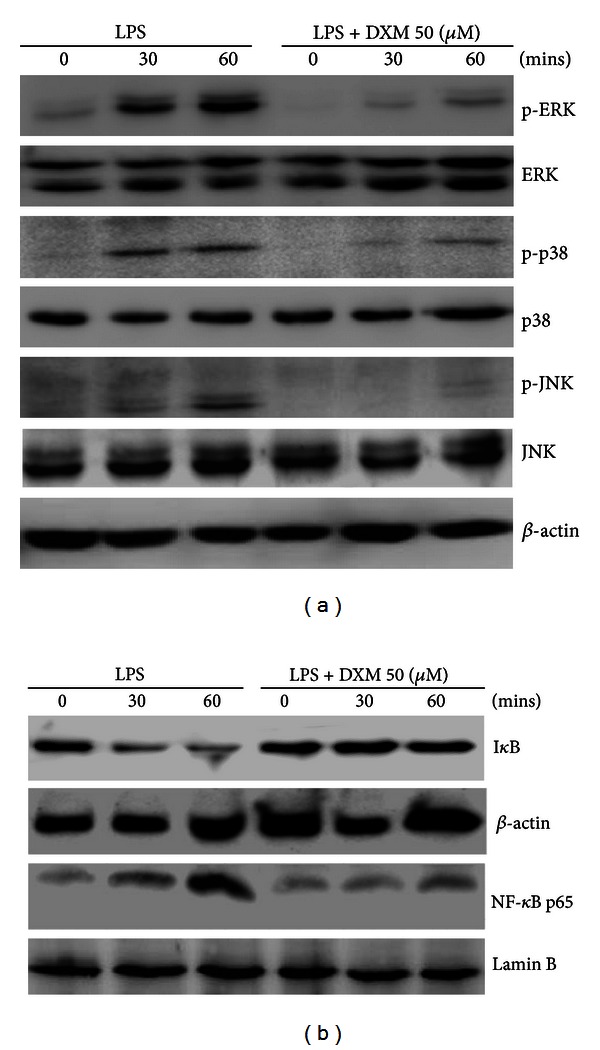
DXM inhibition of MAPK and NF-*κ*B activation in BMDCs. Immature BMDCs were stimulated with 100 ng/mL LPS with or without DXM and lysed at the indicated time points. (a) The ERK, JNK, and p38 MAPK (native and phosphorylated) in whole cell lystes. (b) I*κ*B*α* in whole-cell lystes and NF-*κ*B p65 in nuclear extracts as mentioned were determined by Western blot with antibodies. *β*-actin and Lamin B loading controls are also shown to demonstrate relatively equal protein load across all lanes. The data are representative of three independent experiments showing similar results.

Because the critical function of mature DCs is to activate T-cell proliferation, we determined whether DXM-treated BMDCs could induce antigen-specific CD4^+^ and CD8^+^ T-cell responses. OVA_257−264_ (OVAP_1_) or OVA_323−339_ (OVAP_2_) peptide-loaded immature BMDCs were preincubated in the presence or absence of DXM, stimulated with LPS, and tested for their ability to stimulate allogeneic OVA-specific CD4^+^ OT-II or CD8^+^ OT-I T cells. T-cell proliferation was measured by [^3^H] thymidine incorporation. Coculture with LPS-stimulated BMDCs effectively enhanced CD4^+^ OT-II and CD8^+^ OT-I T-cell proliferative responses, but this proliferation was reduced by DXM (Figure 4). Because IFN-*γ* is produced by activated T cells, IFN-*γ* in the culture supernatants was measured using ELISA. As shown in Figure 5, DXM treatment reduced the IFN-*γ* produced by activated CD4^+^ and CD8^+^ T cells. Thus, DXM attenuated the ability of DCs to activate Ag-specific T-cell immune responses. 

### 3.4. DXM Suppressed MAPK and NF-*κ*B Pathways in LPS-Stimulated BMDCs

The activation of MAPKs and NF-*κ*B is crucial for DC maturation and the inflammatory response [26]. The LPS stimulation of TLR-4 signaling activates MAPKs and NF-*κ*B signal pathways, resulting in DC maturation [27, 28]. To explore the molecular mechanisms of the DXM inhibitory effect, we determined whether MAPKs and NF-*κ*B activation were altered by DXM in LPS-stimulated BMDCs. DXM treatment blocked the phosphorylation of MAPKs ERK, p38, and JNK but did not affect the level of unphosphorylated proteins (Figure 5(a)). To determine whether DXM decreased NF-*κ*B activation, the expression of I*κ*B protein and nuclear translocation of NF-*κ*B p65 were measured. I*κ*b is known to be an inhibitor of NF-*κ*b and can form a complex with the NF-*κ*b, thereby preventing nuclear translocation of NF-*κ*b. Under partial external stimulus such as LPS, I*κ*B undergoes phosphorylation and degradation, thereby unlocking NF-*κ*b and resulting in the nuclear translocation of NF-*κ*b and the activation of related signaling pathways. As shown in Figure 5, in LPS-stimulated BMDCs, DXM treatment prevented downregulation of I*κ*B*α* protein (Figure 5(a)) and decreased NF-*κ*B p65 nuclear localization (Figure 5(b)). These results suggest that DXM inhibits LPS-induced DC activation, possibly by disrupting the MAPK and NF-*κ*B pathways.

### 3.5. DXM Affects the Expression of Surface Markers and Cytokine Secretion in Human Monocyte-Derived DCs (MDDCs)

**Figure 6:**
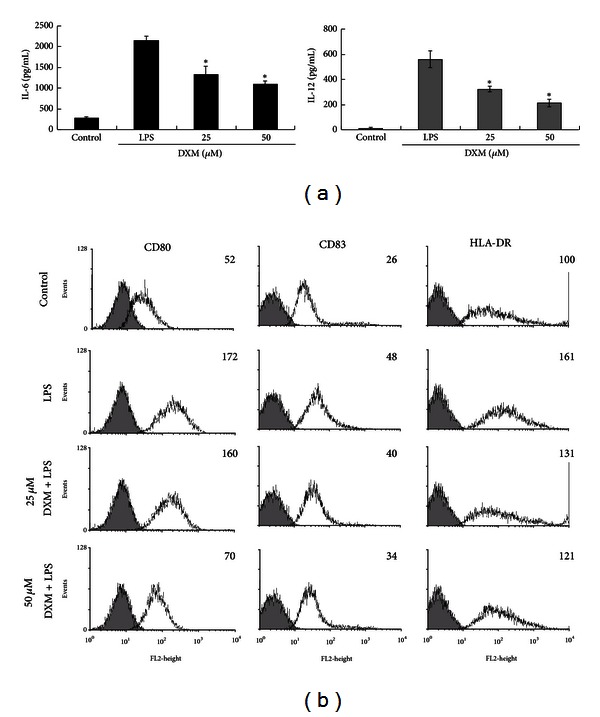
DXM inhibited human MDDC activation. Immature MDDCs were treated with LPS (100 ng/mL) + IFN-*γ* (10 ng/mL), LPS (100 ng/mL) + IFN-*γ* (10 ng/mL) + DXM (25, 50 *μ*M) for 18 h. The control group was treated with PBS alone. (a) Supernatants were collected 18 h later (TNF, 6 h), and TNF-alpha, IL-6, and IL-12 production was measured by ELISA. Data are presented as the mean ± SD of samples from three wells. Significant differences between DXM-treated and untreated LPS + IFN-*γ*-activated DCs are shown with asterisks (*P < 0.05). (b) The expression of CD80, CD83, and HLA was determined by flow cytometry. All data were gated on CD1a^+^ cells. The gray-filled area represents staining with an isotype-matched control Ab. The change of geometric mean fluorescence intensity in the LPS + IFN-*γ* or LPS + IFN-*γ* + DXM samples is indicated. All data are representative of five independent experiments with cells from individual donors.

In addition to murine BMDCs, we examined whether DXM regulates LPS-induced surface molecule expression and cytokine production in human MDDCs. MDDCs were cultured in the presence or absence of DXM for 1 h and then stimulated with LPS (100 ng/mL) plus IFN-*γ* (10 ng/mL). Immature MDDCs stimulated with LPS plus IFN-*γ* released IL-6 and IL-12. The release of these cytokines was suppressed by incubation with DXM (Figure 6(a)). We also analyzed the effect of DXM on the expression of DC surface activation markers. The LPS stimulation of MDDCs resulted in the upregulation of CD80, CD83, and HLA-DR; however, this upregulation was significantly inhibited by DXM (Figure 6(b)). Also, these inhibited effects were not due to cytotoxicity of DXM, because there were no marked difference in the cell viability and percentage of Annexin V^+^/dead cells in cultures containing DXM or PBS-treated controls (Supplemental Figure 1(b)).

## 4. Discussion

Because DCs can initiate primary T-cell responses, they form a crucial interface between innate and adaptive immunity. Potential interference with this essential cell type might affect the pharmacological profile of an immunosuppressive drug [10–14]. In this study, we examined the activity of DXM, a widely used antitussive, on the immune function of DCs. We showed that DXM interfered with DC maturation, as measured using costimulatory molecules, cytokine, reactive oxygen species (ROS), and stimulation of allogeneic T cells. This is the first study to report that DXM has an immunomodulatory effect on DCs.

The NF-*κ*B signaling pathway is critical for DC maturation and cytokine production [28]. The NF-*κ*B signaling pathway includes several important molecules such as NF-*κ*B, I*κ*B, and I*κ*B kinase [29]. DXM inhibits LPS-induced I*κ*B*α* degradation and the nuclear translocation of p65 in human endothelial cells [30]. MAPK signaling pathways have also received attention as molecular targets for DC therapies [26–28, 31, 32]. The minimal MAPK cascade consists of a three kinase core where an MAP3 K (MAP2 K kinase) activates a MAP2 K (MAPK kinase) that activates an MAPK (ERK, JNK, p38), resulting in the activation of NF-*κ*B pathways that contribute to cell growth, survival, and antiapoptosis [33]. In this study, we showed that DXM decreased NF-*κ*B and MAPK (ERK, p38, JNK) activation in LPS-treated BMDCs (Figure 5), and this inhibitory effect was associated with DC maturation. 

Reactive oxygen species (ROS) are also known to have important signaling properties, including activation of NF-*κ*B and MAPK signaling in many cell types [34]. ROS are also known to influence the production and secretion of cytokines; after exposure to ROS, DCs more efficiently present antigens [25]. Previous studies have shown that DXM has antioxidant properties in many cell types [16, 19, 35–37]. In this study, we investigated whether DXM could affect ROS formation during the process of LPS-stimulated dendritic cell maturation. Our results confirmed that DXM inhibited ROS production in LPS-stimulated BMDCs. Although the underlying mechanism remains unclear, suppressed ROS production due to the inhibition of NOX2, iNOS, or NADPH oxidase expression and activity is possible [16, 19, 35, 38]. NOX2- and NADPH oxidase-deleted dendritic cells cannot be induced to mature [39, 40]. Further investigation of the influence and possible mechanism of action of DXM on NOX2 and NADPH oxidase in dendritic cells is necessary.

Because activated DCs regulate T-cell responses, the type of cytokines that they release may determine whether CD4^+^ T cells mature into Th1, Th2, Th17, or Treg cells [41]. IL-12 drives T helper type 1 (Th1) responses, whereas IL-4 promotes Th2-type responses [42]. We observed that DXM significantly inhibited LPS-induced IL-12 production in murine and human DCs (Figures 2 and 6). In addition, we showed that LPS-stimulated OVA peptide-pulsed BMDCs skewed naive OT-II T cells toward IFN-*γ*-producing T cells, but OT-II T cells stimulated with OVA-pulsed BMDCs exposed to DXM produced lower levels of IFN-*γ* (Figure 4). Because IFN-*γ* is a major product of Th1 cells [43], these results suggest that DXM may be effective in several Th1-dominant chronic inflammatory diseases, such as multiple sclerosis (MS), diabetes, and rheumatoid arthritis (RA) [44].

The present study used the TLR-4 ligand LPS to stimulate DC maturation. LPS induces strong Th1-like responses but not Th2 immune responses [45]. We did not observe IL-4 expression in DCs after LPS stimulation (data not shown). However, we cannot exclude the possibility that DXM affects Th2 responses. Therefore, substances capable of stimulating Th2 immune responses, such as dust mite allergens [46], should be used in future investigations of the effects of DXM on DC-mediated Th2 responses.

We also found that DXM suppressed TNF-alpha expression when it was given before or after LPS stimulation (Supplemental Figure 2), implying that the anti-inflammatory and immunomodulatory effects of DXM could be used for prevention or treatment purposes. Although LPS was the main stimulus used for DC maturation in this study, we also tested whether DXM could modulate the activation of immature BMDCs by other TLR ligands and applied Pam3CSK4, PolyI:C, flagellin, and CpG ODN ligands for TLR1/TLR2, TLR3, TLR5, and TLR9, respectively. The presence of each substance resulted in the release of the proinflammatory cytokine TNF-alpha. This release was completely inhibited by 50 *μ*M of DXM (Supplemental Figure 3). Although the mechanism of DXM interference with DC activation after TLR ligand stimulation is not entirely clear, we suggest that it may be related to the inhibitory effect of DXM on MAPK and NF-*κ*B activation. NF-*κ*B is required for DCs to secrete inflammatory cytokines after they are stimulated with various TLR ligands [29]. Further investigation of the effect of DXM on DC maturation through other non-TLR pathways such asflt3 or c-kit ligands [47] or GM-CSF, IL-1*β*, and IL-7 (FKGm17) cytokine stimuli is necessary.

Increased IDO expression in DCs can cause T-cell apoptosis via tryptophan starvation [48]. IDO expression in DCs may be related to the differentiation of Treg cells [49, 50]. We found that DXM at 50 *μ*M did not induce IDO expression or alter LPS-induced IDO expression (Supplemental Figure 4(a)). Previous studies reported that IL-10 inhibits effector T-cell responses and may induce Tr1 regulatory T-cell differentiation [51, 52]. In this study, ELISA indicated that no significant alteration in IL-10 expression was found in BMDCs treated with or without LPS (Supplemental Figure 4(b)). Based on these results, we suggest that the T-cell-inhibitory effect of DXM might occur via the suppression of surface costimulatory receptor expression and cytokine release. Further analysis of more immunomodulatory factors, such as high levels of PD-L1 (programmed death-1 ligand), retinoic acid (RA), TGF-beta, or other factors capable of activating Treg differentiation or activation is required [53, 54].

The clinical dose of DXM for adult human is 60–120 mg/kg/day, and the peak concentration of DXM is about 8–16 *μ*M in serum after administration [55]. Another report describes that the maximum concentration of DXM is about 0.8–9.64 mg/kg (as high as 2 *μ*M) in the serum of neurosurgery patients; however, the concentration of DXM in brain can be 68-fold higher than that in serum [56]. In our in vitro study, we found that 12.5 *μ*M–50 *μ*M DXM can attenuate the LPS-induced murine and human DC activation, which dosage is possible in physiological condition, suggesting that DXM may have a potential to modulate DC function in vivo. 

## 5. Conclusion

In summary, we provided evidence for a novel cellular target of DXM in alloimmune responses in addition to its well-known T-cell inhibitory capacity. Because of its potent effects on DCs, DXM may potentially prevent or treat DC-associated chronic or acute immune diseases, such as MS, diabetes, and RA [44]. Because DCs are important for the eradication of tumors and pathogens [57–59], future clinical studies should identify the risks associated with long-term DXM use. 

## Supplementary Material

Supplemental Figure 1: DXM cytotoxicity in DCs.
The result showed that there was no marked difference in the percentage of dead cells in cultures containing 100100 **µ**M DXM or PBS treated controls which suggested that DXM did not have any cytotoxicity in DCs.
Supplemental Figure 2: DXM treatment before or after LPS stimulation impaired TNF-alpha an IL-12 production in mBMDCs.
The result showed that DXM suppressed TNF-alpha and IL-12 expression when it was given before or after LPS stimulation, implying that the anti-inflammatory and immunomodulatory effects of DXM could be used for prevention or treatment purposes.
Supplemental Figure 3: DXM impaired TNF-alpha production in mBMDCs stimulated by various TLR ligands.
We tested whether DXM could modulate the activation of immature DCs by other TLR ligands and applied Pam3CSK4, PolyI:C, flagellin, and CpG ODN ligands for TLR1/TLR2, TLR3, TLR5, and TLR9, respectively. The presence of each substance resulted in the release of the proinflammatory cytokine TNF-alpha. This release was completely inhibited by 50 **µ**M of DXM.
Supplemental Figure 4: DXM did not alter IDO and IL-10 expression in LPS+IFN–*γ*-treated or untreated mBMDC cells.
A) The result showed that DXM at 50 **µ**M did not induce IDO expression or alter LPS-induced IDO expression. In addition, previous studies reported that IL-10 inhibits effector T-cell responses and may induce Tr1 regulatory T-cell differentiation [52,53]. In this study, B) ELISA indicated that no significant alteration in IL-10 expression was found in DCs treated with or without LPS.
