# Supplementary figures and images for: Dextromethorphan Inhibits Activations and Functions in Dendritic Cells
Source: Clin Dev Immunol. 2013 May 28;2013:125643. doi: 10.1155/2013/125643 (PMC3679715; doi:10.1155/2013/125643)

**Supplemental Figure 1**

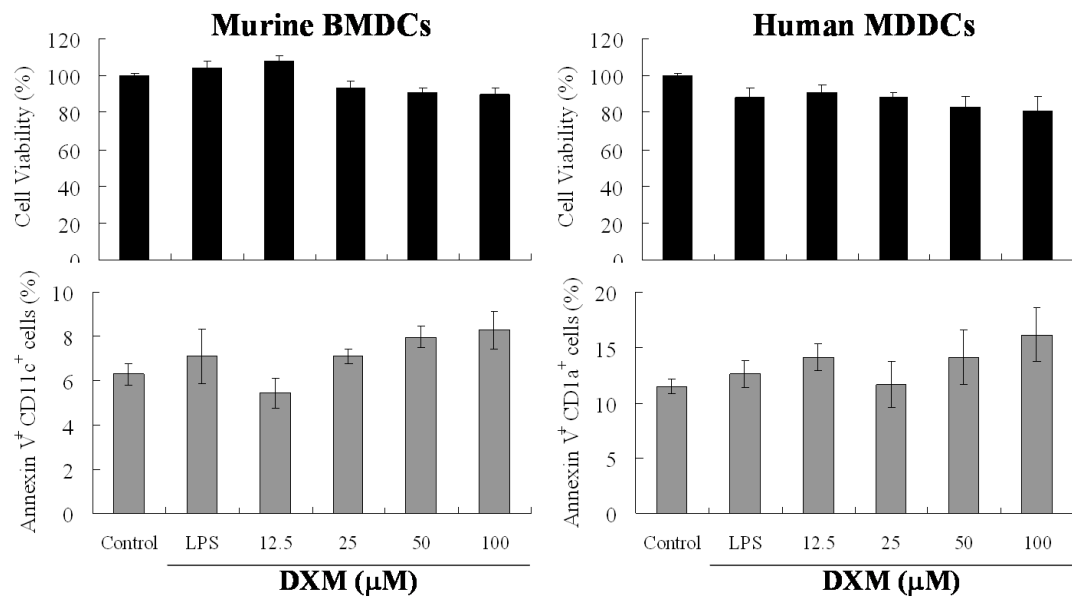

**Supplemental Figure 2**

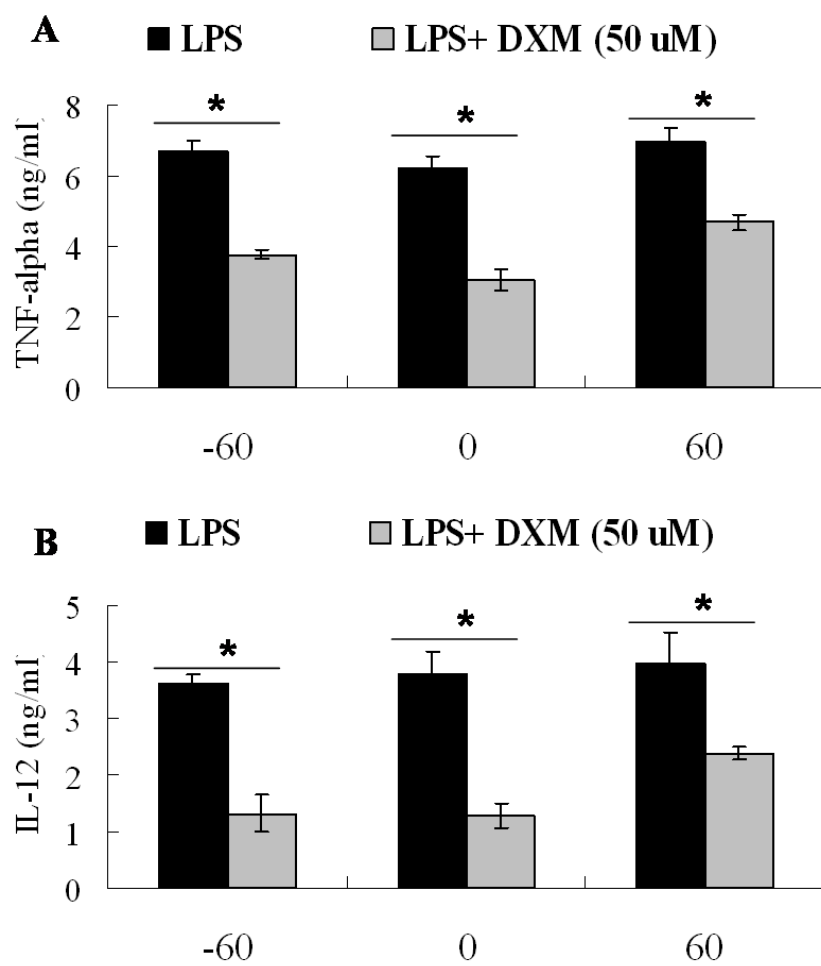

**Supplemental Figure 3**

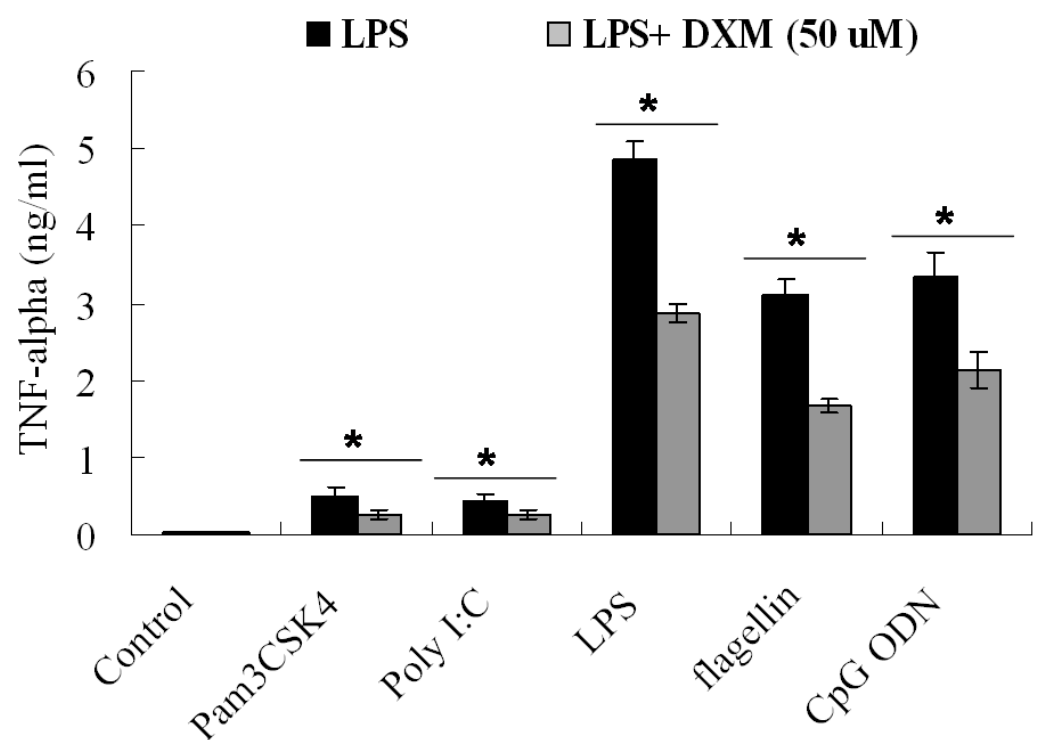

Supplemental Figure 4

**A**

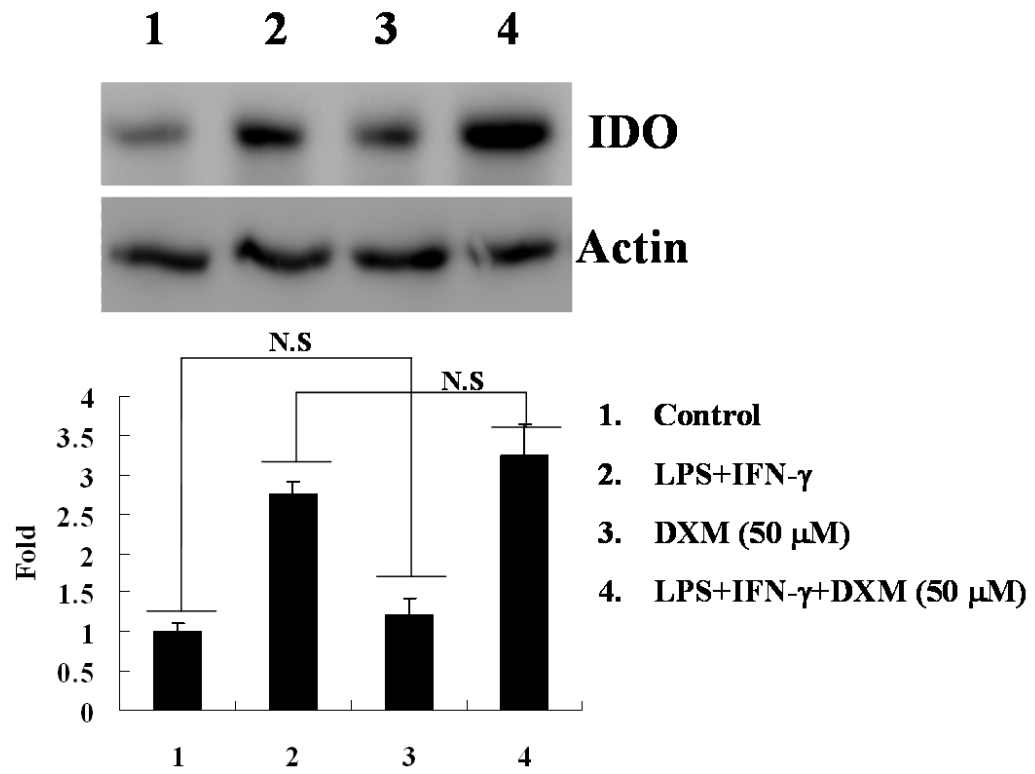

**B**

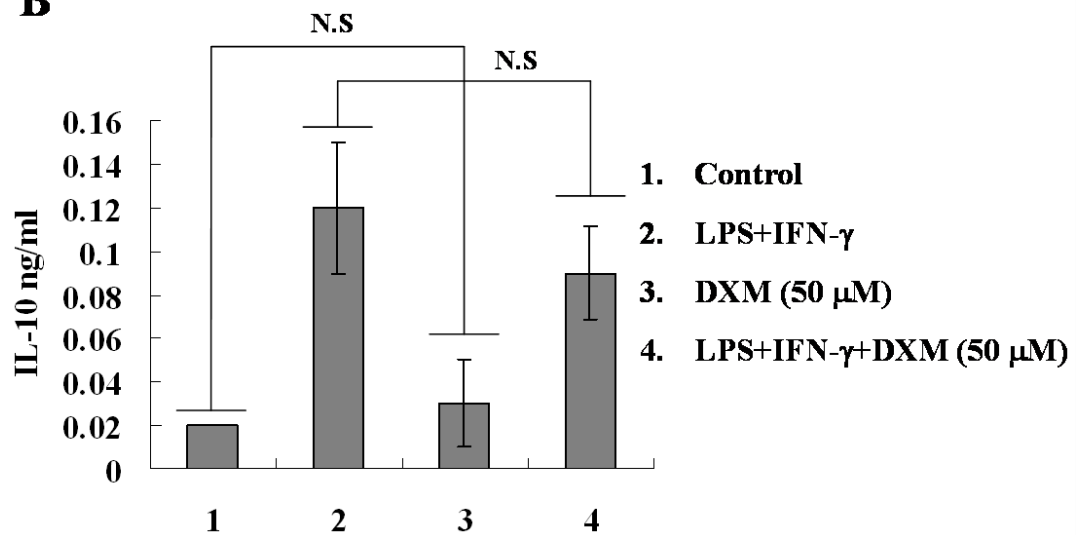

Supplement: Supplementary file 1 — Supplemental Figure 1: DXM cytotoxicity in DCs. The result showed that there was no marked difference in the percentage of dead cells in cultures containing 100100 µM DXM or PBS treated controls which suggested that DXM did not have any cytotoxicity in DCs. Supplemental Figure 2: DXM treatment before or after LPS stimulation impaired TNF-alpha an IL-12 production in mBMDCs. The result showed that DXM suppressed TNF-alpha and IL-12 expression when it was given before or after LPS stimulation, implying that the anti-inflammatory and immunomodulatory effects of DXM could be used for prevention or treatment purposes. Supplemental Figure 3: DXM impaired TNF-alpha production in mBMDCs stimulated by various TLR ligands. We tested whether DXM could modulate the activation of immature DCs by other TLR ligands and applied Pam3CSK4, PolyI:C, flagellin, and CpG ODN ligands for TLR1/TLR2, TLR3, TLR5, and TLR9, respectively. The presence of each substance resulted in the release of the proinflammatory cytokine TNF-alpha. This release was completely inhibited by 50 µM of DXM. Supplemental Figure 4: DXM did not alter IDO and IL-10 expression in LPS+IFN–γ-treated or untreated mBMDC cells. A) The result showed that DXM at 50 µM did not induce IDO expression or alter LPS-induced IDO expression. In addition, previous studies reported that IL-10 inhibits effector T-cell responses and may induce Tr1 regulatory T-cell differentiation [52,53]. In this study, B) ELISA indicated that no significant alteration in IL-10 expression was found in DCs treated with or without LPS. [file 125643.f1.pdf]
